# Self-attention random forest for breast cancer image classification

**DOI:** 10.3389/fonc.2023.1043463

**Published:** 2023-02-06

**Authors:** Jia Li, Jingwen Shi, Jianrong Chen, Ziqi Du, Li Huang

**Affiliations:** Department of Intelligent Manufacturing, Wuyi University, Jiangmen, China

**Keywords:** classification, breast cancer images, self-attention, random forest, GridSearchCV

## Abstract

**Introduction:**

Early screening and diagnosis of breast cancer can not only detect hidden diseases in time, but also effectively improve the survival rate of patients. Therefore, the accurate classification of breast cancer images becomes the key to auxiliary diagnosis.

**Methods:**

In this paper, on the basis of extracting multi-scale fusion features of breast cancer images using pyramid gray level co-occurrence matrix, we present a Self-Attention Random Forest (SARF) model as a classifier to explain the importance of fusion features, and can perform adaptive refinement processing on features, thus, the classification accuracy can be improved. In addition, we use GridSearchCV technique to optimize the hyperparameters of the model, which greatly avoids the limitation of artificially selected parameters.

**Results:**

To demonstrate the effectiveness of our method, we perform validation on the breast cancer histopathological image-BreaKHis. The proposed method achieves an average accuracy of 92.96% and a micro average AUC value of 0.9588 for eight-class classification, and an average accuracy of 97.16% and an AUC value of 0.9713 for binary classification on BreaKHis dataset.

**Discussion:**

For the sake of verify the universality of the proposed model, we also conduct experiments on MIAS dataset. An excellent average classification accuracy is 98.79% on MIAS dataset. Compared to other state-of-the-art methods, the experimental results demonstrate that the performance of the proposed method is superior to that of others. Furthermore, we can analyze the influence of different types of features on the proposed model, and provide theoretical basis for further optimization of the model in the future.

## 1 Introduction

At present, breast cancer has become a major health problem worldwide and the second most common cause of female cancer death (1), especially in Asia, Africa, South America and other regions where the incidence of pre-breast cancer is low, the growth rate is particularly obvious (2). To a certain extent, experienced physicians can complete the diagnosis by discriminating histopathological images. However, this traditional diagnostic approach has certain subjectivity, low diagnostic efficiency and can’t be repeated. Therefore, computer aided diagnosis (CAD) has been widely used (3–5), and it has achieved reliable results both in classifying a large amount of breast cancer data by algorithms or in predicting new data based on previous data. It has also been recognized and trusted by the majority of histopathologists and greatly reduced the workload of doctors.

Image feature extraction or feature selection is an important prerequisite for building high quality breast cancer diagnostic models, so how to extract features from images that are useful for recognition has been a concern of researchers. For example, the literature (6) extracted features from the gray-scale map of breast cancer histopathological images using gray-level co-occurrence matrix (GLCM), local bimary patterns (LBP), law’s texture energy (LTE) and Haralick texture feature (HTF) feature extraction methods; literature (7) used color-texture features to describe the image with Gabor features, multi-layer coordinate cluster representation, etc.; literature (8) extracted features from three aspects: morphological features, spatial features and texture features. In addition, how to combine certain feature extraction methods to construct effective classification model is also the focus of attention. For example, Spanhol F A et al. (4) adopted completed local binary pattern(CLBP), GLCM and parameter-free threshold adjacency statistics (PFTAS) as feature extraction means, which were applied to different classifiers such as random forest (RF), K-Nearest Neighbor (KNN) and support vector machine (SVM), experimental results showed that the combination of PFTAS and SVM classifier achieved better performance with 85% accuracy; Vartika Mishra et al. (9) used scale-invariant feature transform (SIFT) and speeded up robust features (SURF) descriptor feature extraction techniques were used to extract features from breast cancer histopathological images, followed by dimensionality reduction using principal component analysis (PCA), and the performance of the four classifiers was analyzed objectively, the results shows that KNNhas the highest accuracy among SIFT, SIFT-PCA, SURF and SURF-PCA, and SURF is faster than SIFT. Although various machine learning models constructed above have made efforts in the classification accuracy, the clinical results have higher requirements forCAD. Therefore, we still need to explore better methods to improve the classification effect of breast cancer images.

Deep learning (DL) has been successfully used to accomplish breast cancer histopathological image recognition tasks (10–14). For example, Kassani S H et al. (15) proposed an integrated model of convolutional neural network (CNN) based on VGG19, MobileNet and DenseNet for feature representation and extraction steps, and the proposed integrated model obtained better predictions than single classifier and machine learning algorithms. In addition, in recent years, as Volodymyr (16) applied attention mechanism to the visual field for the first time in 2014 and made a great breakthrough, more and more researchers introduced attention mechanism into breast cancer image recognition task (17–20), which confirmed that attention mechanism can increase the expression of features. It makes the classifier pay more attention to important features and suppress unimportant features. Although the aforementioned deep learning techniques have shown remarkable results in image classification, there are still certain problems, firstly, the complex structure of deep learning and the involvement of a large number of convolutional operations lead to a large training time cost, secondly, the poor interpretability of the deep model makes it difficult to design an effective optimization strategy for neural network. To address these problems, the traditional machine learning (4, 9) model has been shown to be effective structures. To further improve the performance of breast cancer image recognition, inspired by the literature (21) and Self-Attention Network (SAN) (22), we propose a self-attention random forest (SARF) model based on a common idea (extracting multiscale features and focusing on important features), and combine with the advantages of random forest such as simple structure and interpretability. The main contributions of our work are as follows:

1) We introduce the self-attentive mechanism into the classification model, SARF can realize the adaptive refinement of multi-scale features, which makes the model pay more attention to the learning of important features.
2) Using the model interpretable SHAP (Shapley Additive exPlanations), we analyze the degree of influence of each scale feature in the sample on the prediction results of SARF model, which plays an active role in the subsequent model optimization.
3) Our overall framework consists of feature extraction and classification. In the feature extraction stage, the multi-scale fusion features of breast cancer images are extracted using pyramid gray level co-occurrence matrix (PGLCM); in the classificationstage, the GridSearchCV technique is used to optimize the classification ability of SARF model, which effectively avoids the limitation of artificial parameter selection.
4) Experiments show that, compared with the existing advanced algorithms, the performance of the proposed method has been significantly improved on histopathological image dataset (BreakHis). In addition, the proposed method is also applicable to mammographic dataset (MIAS), which reflects the universality of the model.

The rest of the paper is organized as follows: the datasets and evaluation metrics are presented in Section 2. Our proposed method is presented in Section 3. In Section 4, we describe the details of the experiments and the results. The paper is discussed in Section 5. At last, we make a brief conclusion about this paper in Section 6.

## 2 Datasets and evaluation metrics

### 2.1 Datasets

#### 2.1.1 BreaKHis

The BreaKHis dataset (4) from the P&D laboratory—Pathological Anatomy and Cytopathology in Paraná, Brazil, which consists of hematoxylin and Eosin (H&E)-stained histopathological images of breast cancer from 82 patients (24 benign and 58 malignant), with a total of 7909 images (3-channel RGB, 8-bit depth per channel, 700 × 460 pixels, PNG format), containing 5429 malignant tumor samples and 2480 benign tumor samples.

**Figure 1:**
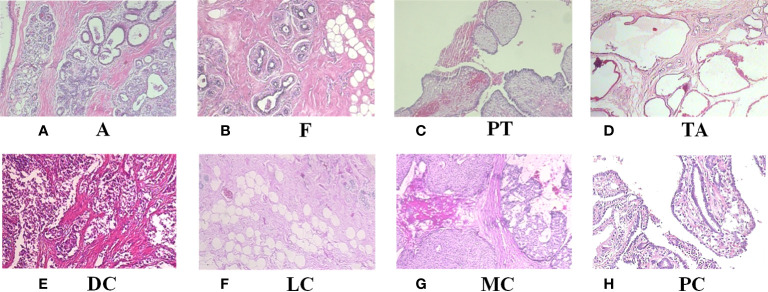
H&E-stained breast cancer histopathological images of 8 tumor types. **(A)** A, adenosis; **(B)** F, fibroadenoma; **(C)** PT, phyllodes tumor; **(D)** TA, tubular adenoma; **(E)** DC, ductal carcinoma; **(F)** LC, lobular carcinoma; **(G)** MC, mucinous carcinoma; **(H)** PC, papillary carcinoma.

**Table 1** shows the contents of BreaKHis dataset, which contains images of benign tumors (B) and malignant tumors (M) at 4 different magnifications (40X, 100X, 200X, 400X), with B including adenosis (A), fibroadenoma (F), phyllodes tumor (PT), and tubular adenoma (TA), while the M include ductal carcinoma (DC), lobular carcinoma (LC), mucinous carcinoma (MC) and papillary carcinoma (PC), a total of 8 types. **Figure 1** shows the H&E-stained breast cancer histopathological images for these 8 tumor types, where (a)(b)(c)(d) is B, (e)(f)(g)(h) is M.

**Table 1:** Summary of the contents of BreaKHis.

Magnification	Benign				Malignant				Total
	A	F	PT	TA	DC	LC	MC	PC	
40X	114	253	109	149	864	156	205	145	1995
100X	113	260	121	150	903	170	222	142	2081
200X	111	264	108	140	896	163	196	135	2013
400X	106	237	115	130	788	137	169	138	1820
Total	444	1014	453	569	3451	626	792	560	7909
Patients	4	10	3	7	38	5	9	6	82

A, adenosis; F, fibroadenoma; PT, phyllodes tumor; TA, tubular adenoma; DC, ductal carcinoma; LC, lobular carcinoma; MC, mucinous carcinoma; PC, papillary carcinoma.

#### 2.1.2 MIAS

The MIAS dataset (23) from the UK national breast screening program (UK NBSP), consists of 322 left and right breast mammographic images from 161 patients (grey-scale images, 1024 × 1024 pixels, PGM format), classified into 3 categories: normal (207), benign (63) and malignant (52). The dataset also distinguishes between images labeled as benign and malignant tumor images for background tissue and etiology, with background tissue divided into 3 categories: fatty, fatty-glandular and dense-glandular; etiology divided into calcification (CALC), well-defined/circumscribed masses (CIRC), spiculated masses (SPIC), miscellaneous/ill-defined masses (MISC), architectural distortion (ARCH), asymmetry (ASYM). **Table 2** shows the contents of the MIAS datasets.

**Table 2:** Summary of the contents of MIAS.

Film Category	Breast Type			Total	
	Fatty	Fatty-Glandular	Dense-Glandular		
CALC	B: 2	B: 5	B: 5	B: 12	25
	M: 4	M: 4	M: 5	M: 13	
CIRC	B: 10	B: 6	B: 3	B: 19	23
	M: 2	M: 2	M: 0	M: 4	
SPIC	B: 2	B: 4	B: 5	B: 11	19
	M: 3	M: 3	M: 2	M: 8	
MISC	B: 2	B: 3	B: 1	B: 6	14
	M: 5	M: 2	M: 1	M: 8	
ARCH	B: 4	B: 2	B: 3	B: 9	19
	M: 2	M: 4	M: 4	M: 10	
ASYM	B: 1	B: 2	B: 3	B: 6	15
	M: 3	M: 2	M: 4	M: 9	
Normal	66	65	76	207	207
Total	106	104	112	322	322

B, benign; M, malignant; CALC, calcification; CIRC, well-defined/circumscribed masses; SPIC, spiculated masses; MISC, miscellaneous/ill-defined masses; ARCH, architectural distortion; ASYM, asymmetry.

**Figure 2:**
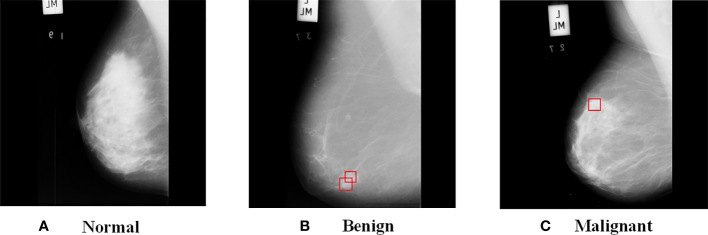
Different types of images in MIAS dataset. **(A)** Normal; **(B)** Benign; **(C)** Malignant.

**Figure 2** shows the images from MIAS dataset, where (a) is a normal breast image, (b) is a benign tumor breast image, where the red box indicates a mass with fatty background tissue and etiology is CIRC, and (c) is a malignant tumor breast image, where the red box indicates fatty background tissue and etiology is ASYM. As can be seen from the **Figure 2**, benign tumors have smooth edges and regular shapes, while malignant tumors have blurred edges and burrs.

### 2.2 Evaluation metrics

For the selection of evaluation metrics, the confusion matrix as well as Accuracy (Acc)[37], sensitivity (Sen), specificity (Spe), positive prediction rate (PPR), negative prediction rate (NPR), receiver operating characteristic (ROC) curve and area under curve (AUC) as evaluation metrics, and the larger values of Acc, Sen, Spe, PPR and NPR represent the closer the model’s prediction and the real situation, the better the model performance, and their expressions as follows:


(1) $$Acc=\sum_{i=1}^{n}N_{ii}/\sum_{i=1}^{n}\sum_{j=1}^{n}N_{ij}$$

(2) $$Sen=\frac{TP}{TP+FN}$$

(3) $$Spe=\frac{TN}{TN+FP}$$

(4) $$PPR=\frac{TP}{TP+FP}$$

(5) $$NPR=\frac{TN}{TN+FN}$$

Where *TP* denotes true positive cases, *FN* denotes false negative cases, *FP* denotes false positive cases, and *TN* denotes true negative cases, *n* is the number of categories, *N*_*ij*_ represents the value of row extiti and column *j* in the confusion matrix.

The ROC curve takes the false positive rate (FPR) as the horizontal coordinate and the true positive rate (TPR) as the vertical coordinate, and the AUC value is the area under the ROC curve. The closer the ROC curve is to the upper left corner and the larger the AUC value, the higher the accuracy of the model. The formula of FPR and TPR is defined as:


(6) $$FPR=\frac{FP}{FP+TN}$$

(7) $$TPR=\frac{TP}{TP+FN}$$

## 3 Methods

**Figure 3:**
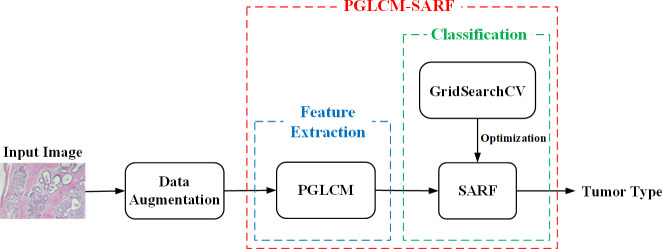
Breast cancer image recognition process.

The proposed process of breast cancer image recognition is shown in **Figure 3**, including three stages of data augmentation, feature extraction and classification, where the feature extraction and classification tasks are performed by the combined model PGLCM-SARF. First, the dataset is expanded and balanced using data augmentation methods such as rotation, flip and enhanced image color degree; second, the features of breast cancer images are extracted using the PGLCM; finally, the SARF model builtas the classifier to complete the breast cancer image recognition task, and the GridSearchCV technique is also used to optimize the classifier.

### 3.1 Data augmentation

As can be seen from **Table 1**, there is an uneven distribution of sample size for different tumor types, especially the sample size of DC type is significantly more than other types. Therefore, in order to expand and balance the data volume, we process the data from BreaKHis and MIAS datasets using data augmentation methods such as rotation and flip, and the sample size distribution after processing is shown in **Tables 3**, **4**.

**Table 3:** The amounts of different types of data in BreaKHis dataset using data augmentation.

Type Tumor Magnification	A	F	PT	TA	DC	LC	MC	PC	Total
40X	912	856	872	894	864	884	905	870	7057
100X	904	891	968	900	903	956	981	851	7354
200X	888	913	864	840	896	918	864	810	6993
400X	848	821	920	780	788	772	746	828	6503
Total	3552	3481	3624	3414	3451	3530	3496	3359	27907

A, adenosis; F, fibroadenoma; PT, phyllodes tumor; TA, tubular adenoma; DC, ductal carcinoma; LC, lobular carcinoma; MC, mucinous carcinoma; PC, papillarycarcinoma.

**Table 4:** The amounts of different types of data in MIAS dataset using data augmentation.

Type	Normal	Benign	Malignant	Total
Total	1569	1575	1560	4704

**Figure 4:**
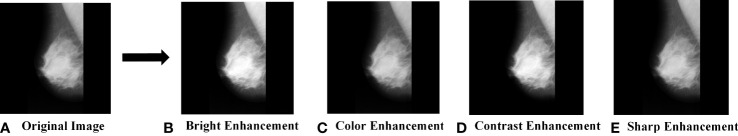
MIAS data augmentation. **(A)** Original Image; **(B)** Bright Enhancement; **(C)** Color Enhancement; **(D)** Contrast Enhancement; **(E)** Sharp Enhancement.

What’s more, to the above data augmentation methods, we also expand and balance the MIAS dataset by enhancing the brightness, chromaticity, contrast, and sharpness of the original images. For example, **Figure 4** shows the enhanced image with an enhancement factor of 1.2 for a benign tumor image.

### 3.2 PGLCM-SARF

#### 3.2.1 PGLCM

**Figure 5:**
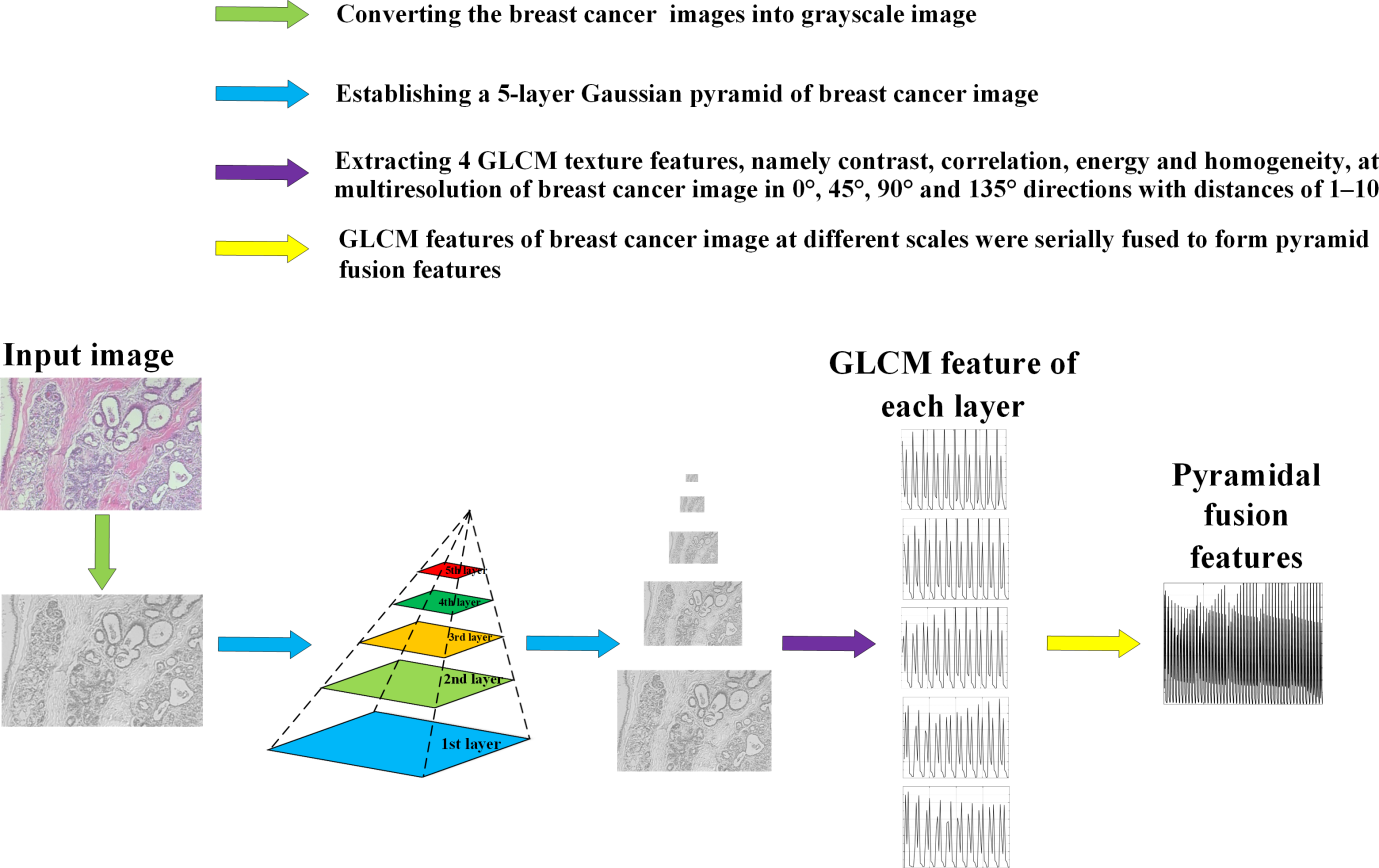
Feature extraction process of breast cancer histopathological images based on PGLCM.

**Figure 5** shows the feature extraction process by using PGLCM (24) with samples from BreaKHis dataset as an example.

**Figure 6:**
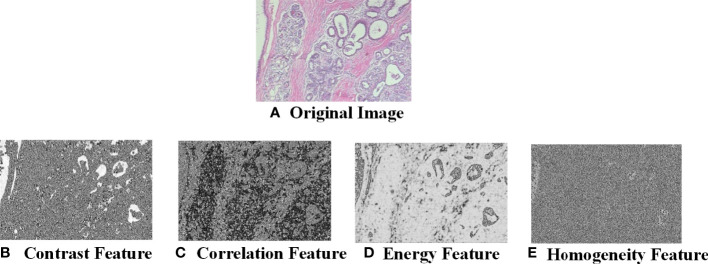
Feature map of breast cancer histopathological image. **(A)** Original Image; **(B)** Contrast Feature; **(C)** Correlation Feature; **(D)** Energy Feature; **(E)** Homogeneity Feature.

**Figure 6** shows the contrast, correlation, energy and homogeneity features map for GLCM of breast cancer histopathological images at a distance of 2 in the 90° direction.

#### 3.2.2 SARF classifier

**Figure 7:**
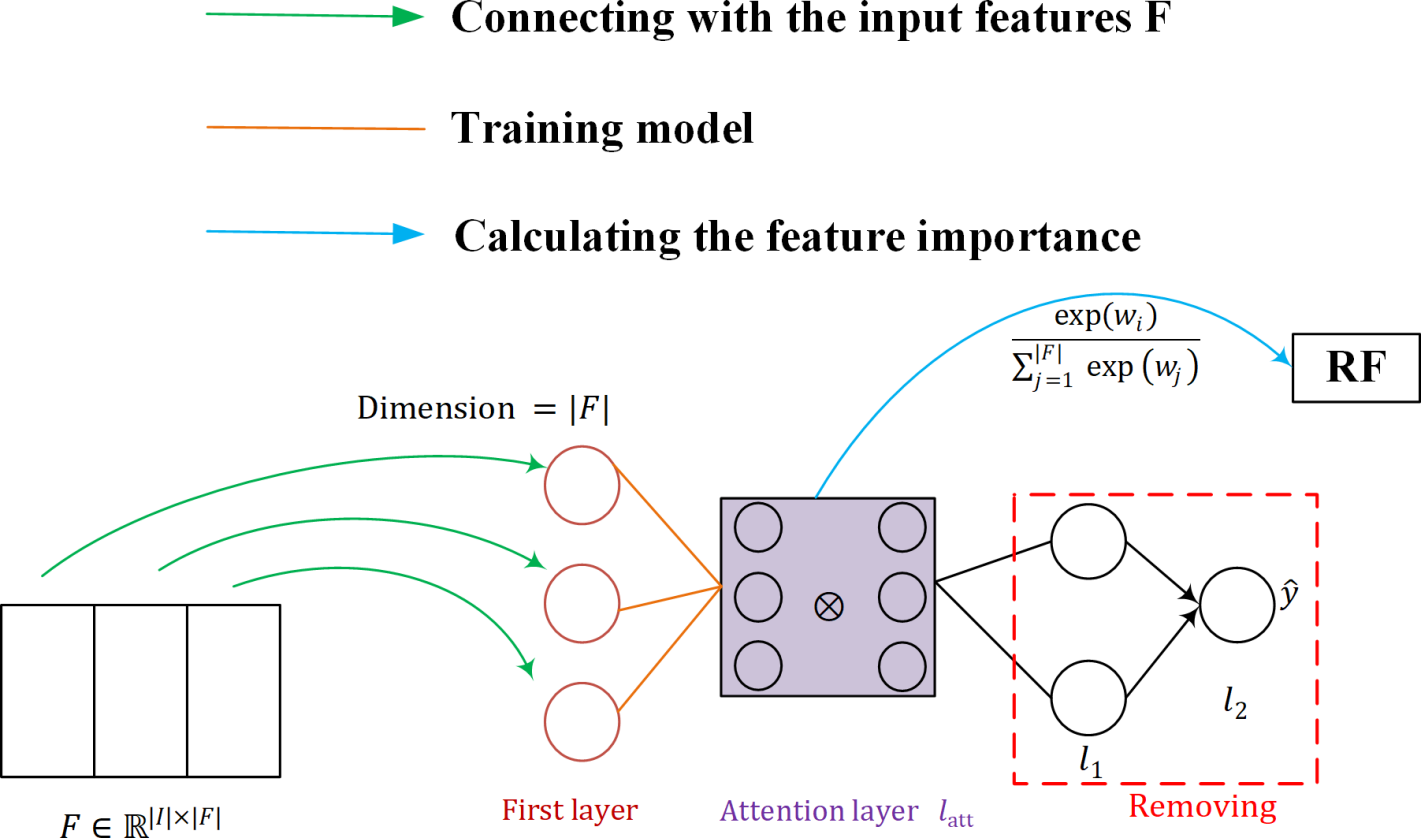
Structure of the SARF proposed. RF, Random Forest.

The structure of the SARF proposed is shown in **Figure 7**. Firstly, the neurons in the first layer of SAN are used to maintain the connection with the input features F (as shown by the green line in **Figure 7**), and then SAN is trained by the attention layer, and the importance W of F is calculated using the trained model (as shown by the blue line in **Figure 7**). Finally, the fused feature vector after weighting W and F is used as the input of the classifier to obtain the classification results. Our purpose of introducing SAN in RF is to take into account the advantages of RF model with its simple structure and interpretability, while achieving important information focus as well as feature adaptive refinement.

The structure of the neural network implementing the attention mechanism can be represented as:


(8) $$l_{2}=\sigma \left(W_{2}\cdot \left(a\left(W_{\left|F\right|}\cdot \text{Ω}\left(X\right)+b_{l_{1}}\right)\right)+b_{l_{2}}\right)$$

The first neural network layer Ω is specifically designed to maintain the connection with the input features *F* . We define it as:


(9) $$\text{Ω}\left(X\right)=\frac{1}{k}{⊕}_{k}\left[X⊗\text{softmax }\left(W_{l_{\text{att}}}^{k}X+b_{l_{\text{att}}}^{k}\right)\right]$$

First, the input vector *X* is used as the input to a softmax activation layer that contains a number of neurons equal to the number of features |*F*| , where the softmax function applied to the.-th element of the weight vector *v* is defined as follows:


(10) $$\text{softmax }\left(v_{j_{i}}\right)=\frac{exp\;\left(v_{j_{i}}\right)}{{\sum^{}}_{j=1}^{\left|F\right|}exp\;\left(v_{j}\right)}$$

where *v*∈*ℝ* ^|*F*|^ and. represents the number of attention heads - the different matrices represent the relationship between the input features. The ⊗ symbol corresponds to the Hadamard product, and ⊕ refers to the Hadamard summation between the individual heads. Thus, Ω represents the first layer of SAN, whose output has dimension |*F*| . a corresponds to the activation function SELU, defined as:


$$\text{SELU }(x)=\lambda \{\begin{array}{ll} x & \text{ if }x>0\\ \alpha \left(\exp\;(x\right)-1) & \text{ if }x\leq 0 \end{array}\left(11\right)$$

where *λ* and *α* are hyperparameters. This method is only interested in self-attention, a SAN is trained and we simply activate the weights of the attention layer by using softmax. In this case, the weight vector itself contains information about the importance of the features and can be checked directly. The global attention is defined as follows:


(12) $$R_{G}=\frac{1}{k}\underset{k}{⊕}\left[\text{softmax }\left(\text{diag }\left(W_{l_{\text{att}}}^{k}\right)\right)\right];W_{l_{\text{att}}}^{k}\in {\mathbb{R}}^{\left|F\right|\times \left|F\right|}$$

SAN is presented below in **Algorithm 1**.

Algorithm 1 SAN corresponding code.
**Input:** X, T, *l*
_2_ , *k* , *α* , *λ*
**Output:**

$W_{l_{\text{all}}}^{k}*\text{ F}+\text{F}$

**initialize**: Set W=0 , *b*=0 **for** *t*=1,2,…,*T* **do** 
$\text{Ω}\left(X\right)=\frac{1}{k}{⊕}_{k}\left[X⊗\text{softmax }\left(W_{l_{\text{all }}}^{k}X+b_{l_{\text{all }}}^{k}\right)\right]$

**if** x> 0 **then** *l*
_2_=sigmoid (*W*
_2_·(*λx*(*W*
_|*F*|_·Ω(*X*)+*b*
_
*l*
_1_
_))+*b*
_
*l*
_2_
_)
**else** *l*
_2_=sigmoid (*W*
_2_·(*λα*(*exp* (*W*
_|*F*|_·Ω(*X*)+*b*
_
*l*
_1_
_)−1))+*b*
_
*l*
_2_
_)
**end if** 
${\hat{y}}_{i}=$
Dense (*l*
_2_) 
$\text{L}=l_{2}\left({\hat{y}}_{i},y\right)$

*L*=*L*−x*dL/*dx*
**update**: *W* , b
**end for**

Random Forest (RF) Breiman (25) is a machine learning algorithm proposed by leo breiman in 2001 by combining the integrated learning theory of bagging with the random subspace method, which is an extended variant of bagging. The basic idea of RF classification: first, n samples are drawn from the original training set using bootsrap sampling (randomly and with put-back from the training set of N training samples), each with the same sample size as the original training set; then, n decision tree models werebuilt for each of the n samples to obtain n classification results; finally, each record is voted on according to the n classification results to determine its final classification.

The RF is interpretable in that it can calculate the feature importance by obtaining the error corresponding to the rearranged feature inputs. The feature importance is defined as *J*_*a*_ with the following equation:


$$J_{a}\left(x_{j}\right)=\frac{1}{T}\sum_{\bar{B_{k}}\in \overset{¯}{C}}\frac{1}{\left|\bar{B_{k}}\right|}\left(\sum_{i\in B_{k}}I\left(h_{k}^{\bar{x_{j}}}\left(i\right)\neq y_{i}\right)-I\left(h_{k}\left(i\right)\neq y_{i}\right)\right)\left(13\right)$$

where *y*_*i*_ denotes the label category corresponding to the i-th Out-Of-Bag (OOB) data, *I*(x) is the indicative function, and *h*_*k*_(*i*) is the function that the label of predict sample *i* . The sample is a sample from dataset *B*_*k*_ and $h_{k}^{rlinex_{j}}\left(i\right)$ is the classification label after replacing feature *x*_*j*_ .

### 3.3 Optimization

Hyperparameters are important for the improvement of a model. RF, as a machine learning algorithm, can be optimized in breast cancer prediction by setting reasonable hyperparameters for the dataset. Hyperparameter optimization is a multivariate function optimization process, commonly used optimization methods are RandomizedSearchCV, HalvingSearchCV, GridSearchCV, Baysian, Gradient-based and so on. In order to automatically obtain the optimal parameters of number and depth of decision trees in RF and avoidthe limitation of human selection, this paper adopts the GridSearchCV algorithm (26) in python library to optimize the parameters of RF. The specific optimization process is as follows: firstly, a grid with a certain numerical interval is given as the search range of parameters in the classification model; then, in the process of training the model, the parameters are selected sequentially in the grid in certain steps, and finally, the parameter with the highest accuracy in multiple iterations is selected as the optimal parameter of the optimized model by cross-validation.

## 4 Experiments and results

This paper validates the effectiveness of the proposed PGLCM-SARF mothed by conducting 3 comparative experiments. First, in order to verify the influence of the introduction of SAN into the classification model on the classification results, our model and PGLCM-RF ablation experiments are conducted (Section 4.1), and in experiment 4.2, analyzing of the ability to improve classification results after optimization of PGLCM-SARF using GridSearchCV techniques. Then, in experiment 4.3, in order to demonstrate the superiority of our method, a comparative experiment is conducted with various current state-of-the-art algorithms. The data used in the aforementioned 3 sets of experiments are all taken from BreaKHis dataset, the adopted data samples are randomly divided into training set (70%) and testing set (30%) in the same proportion. The experiments are done on the Windows 10 64-bit operating system with the following hardware setup: CPU: Intel Core i5-10400, GPU: NVIDIA GeForce GTX 1650,32GB RAM; software platform: MathWorks MATLAB R2018b, python 3.6.

### 4.1 Comparison of PGLCM-SARF and PGLCM-RF

In the parameter setting of PGLCM-SARF, epochs are 20, batch size is 16, learning rate for the Adam optimizer in SAN is set to 0.001, the dropout rate for regularization is set to 20%, and the depth and quantity of decision trees in the RF are set to 20 and 200, respectively. **Table 5** displays the experimental result, our method has a higher classification accuracy than the PGLCM-RF for both binary classification and eight-class classification. An eight-class classification accuracy of 93.72% forPGLCM-SARF and 93.% for PGLCM-RF at a magnification of 100X. Our method has a binary classification accuracy of 97.69%, which is 0.68% better than PGLCM- RF’s. From the results, it can be seen that the introduction of SAN facilitates the adaptive refinement and enhancement of the features and provides a certain enhancement to the classification effect.

**Table 5:** Comparison of accuracy (%) of PGLCM-SARF and PGLCM-RF.

Category	Model	Magnification				
		Irrelevant	40X	100X	200X	400X
Eight-class Classification	Proposed method	**92.67**	**92.79**	**93.72**	**94.16**	**93.67**
	PGLCM-RF	92.63	92.63	93.02	93.96	93.18
	Difference	0.04	0.16	0.70	0.20	0.49
Binary Classification	Proposed method	**97.11**	**97.56**	**97.69**	**97.76**	**97.54**
	PGLCM-RF	96.54	96.94	97.01	97.21	97.17
	Difference	0.22	0.62	0.68	0.55	0.37

The bold values indicate the highest accuracy under the same conditions.

### 4.2 Comparison before and after PGLCM-SARF optimization

We optimize the classification model using the GridSearchCV approach, with parameters set between 190 and 230 for the number of decision trees and 10 to 30 for their depth. **Table 6** compares the average accuracy of the proposed PGLCM-SARF model before and after optimization, bold text denotes RF parameters that are automatically selected (after optimization) using the GridSearchCV approach, and (d, n) stands for the depth and the number of decision trees. In the parameter setting before optimization, we set a fixed (d, n) as (20,200) based on experience. It is clear from **Table 6** that the accuracy rates following optimization have been slightly increased compared to the accuracy rates before to optimization. For instance, in an eight-class classification, the accuracy before optimization ranges from 92.67% to 94.16%, and after optimization, the accuracy ranges from 92.96% to 94.77%, with an improvement of 0.25% to 1.1%. In binary classification, the accuracy before optimization ranges from 97.11% to 97.76%, and after optimization, the accuracy ranges from 97.16% to 97.98%, only 0.05% to 0.29% is different between before and after optimization. The experimental results demonstrate that using the GridSearchCV approach improves classification accuracy. We discover that the higher the accuracy before optimization (that is, the closer to 100%), the smaller the improvement degree after optimization, considering the characteristic of automatic parameter selection of GridSearchCV technology, we think that it is still necessary to adopt this technology.

**Table 6:** Comparison of accuracy before and after GridSearchCV optimization of PGLCM-SARF model.

Category	Magnification	(d, n)	Accuracy (%)	Difference (%)
Eight-class Classification	40X	(20,200)	92.79	1.09
		**(25,223)**	**93.88**	
	100X	(20,200)	93.72	0.25
		**(26,227)**	**93.97**	
	200X	(20,200)	94.16	0.41
		**(24,213)**	**94.57**	
	400X	(20,200)	93.67	1.1
		**(23,219)**	**94.77**	
	Irrelevant	(20,200)	92.67	0.29
		**(26,203)**	**92.96**	
Binary Classification	40X	(20,200)	97.56	0.12
		**(22,218)**	**97.68**	
	100X	(20,200)	97.69	0.29
		**(21,213)**	**97.98**	
	200X	(20,200)	97.76	0.12
		**(27,226)**	**97.88**	
	400X	(20,200)	97.54	0.25
		**(26,197)**	**97.79**	
	Irrelevant	(20,200)	97.11	0.05
		**(26,222)**	**97.16**	

(d, n) represent the depth and number of decision tree, respectively.

The bold values represent optimized parameters and results.

We also utilize the confusion matrix to display the specifics of the classification, which helps to further illustrate the benefits of the improved PGLCM-SARF in terms of recognition impact. The results of each evaluation metrics and confusion matrix are shown in **Table 7** for the optimal binary classification results. In the binary classification experimental test data independent of the magnification, *TP*=3944, *FN*=186, *FP*=52, *TN*=4191, so it can be counted Acc=97.16%, Sen=95.50%, Spe=98.77%, PPR=98.70%, and NPR=95.75%. Take a malignant tumor in the confusion matrix as an example: there are 4130 images in the test set, the number of correctly classified images is 3944, the number of incorrectly classified as benign tumors is 186, and the accuracy of malignant tumor recognition can be calculated as 95.50%. The experimental results show that the recognition accuracy of benign tumor is higher than that of malignant tumor.

**Table 7:** Confusion matrix of binary classification results and values of evaluation metrics.. Sen, sensitivity; Spe, specificity; PPR, positive prediction rate; NPR, negative prediction rate.

Ground Truth	Classification Result		Sen (%)	Spe (%)	PPR (%)	NPR (%)
	Malignant	Benign				
Malignant	3944	186	95.50	98.77	98.70	95.75
Benign	52	4191				

(d, n) = (26, 222).

**Table 8** shows the confusion matrix and the values of the evaluation metrics for the optimal eight-class classification results. In the eight-class classification experimental test data independent of the magnification, taking PC in the confusion matrix as an example: there are 975 images in the test set, the number of correctly classified images is 973, the number of incorrectly classified as DC is 2; the number of incorrectly classified images as other types of tumors is 0, so it can be counted *mathrmSen*_classPC _ =99.79%, Spe_classPC _ =99.15%, PPR_classPC _ =93.92% and NPR_classPC _ =99.97%. Similarly, the results for the remaining 7 categories as shown in **Table 8**, and the average Acc of 92.96% for the last eight-class classes. The results show that A and PT had the highest recognition accuracy, and DC has the lowest, with DC being incorrectly identified as LC the most, indicating that there is some similarity between DC and LC tumor, and the number of DC incorrectly recognized as other tumors is also higher compared with other tumors, indicating that DC tumor have a certain complexity that makes them difficult to identify.

**Table 8:** Confusion matrix of binary classification results and values of evaluation metrics. ((d, n) = (26, 222)). Sen, sensitivity; Spe, specificity; PPR, positive prediction rate; NPR, negative prediction rate; A, Adenosis; F, Fibroadenoma; PT, Phyllodes Tumor; TA, Tubular Adenoma; DC, Ductal Carcinoma; LC, Lobular Carcinoma; MC, Mucinous Carcinoma; PC, Papillary.

Ground Truth	Classification Result								Sen (%)	Spe (%)	PPR (%)	NPR (%)
	PC	MC	LC	DC	TA	PT	F	A				
PC	973	0	0	2	0	0	0	0	99.79	99.15	93.92	99.97
MC	4	1035	3	13	0	2	1	0	97.83	98.59	90.95	99.68
LC	0	0	1043	43	0	0	0	2	95.86	98.17	88.69	99.37
DC	53	92	121	580	49	39	67	49	55.24	99.03	89.09	93.91
TA	0	0	0	3	1005	0	0	0	99.70	99.28	94.99	99.96
PT	0	0	0	0	0	1109	0	0	100.00	99.37	96.02	100.00
F	6	11	9	10	4	5	981	1	95.52	99.07	93.52	99.37
A	0	0	0	0	0	0	0	1058	100.00	99.29	95.32	100.00

(d, n) = (26, 203). Sen, sensitivity; Spe, specificity; Ppr, positive prediction rate; Npr, negartive prediction rate; A, adenosis; F, fibroadenoma; PT, phyllodes tumor; TA, tubular adenoma; DC, ductal carcinoma; LC, lobular carcinoma; MC, mucinous carcinoma; PC, papillary carcinoma.

### 4.3 Comparison with the state-of-the-art methods

In this experiment, firstly, the classification performance of PGLCM-IBL (incremental broad learning) (27) and PGLCM-SARF on breast cancer histopathological images is compared, and secondly, the classification performance of different classification algorithms on breast cancer histopathological images is compared under specific magnification conditions. For the selection of PGLCM-IBL parameters, the number of feature nodes per window is set to 100, the number of enhancement nodes was set to 800, and the number of additional enhanced nodes is set to 10,000; in terms of PGLCM-SARF parameter selection, they are set to the optimal parameters after GridSearchCV optimization (see 4.2).

The experimental results are shown in **Table 9**. The classification accuracy of our method is much higher than that of PGLCM-IBL for both binary classification and eight-class classification, especially in the case of magnification independent. As the results, the binary classification accuracy of our method reaches 97.16%, which is much higher than that of PGLCM-IBL (86.73%); the eight-class classification accuracy of our method is 92.96%, an improvement of 9.53% relative to PGLCM-IBL. The experimental results show that the proposed method has higher classification accuracy for breast cancer histopathological images.

**Table 9:** Comparison of accuracy (%) of PGLCM-SARF and PGLCM-IBL.

Category	Model	Magnification				
		Irrelevant	40X	100X	200X	400X
Eight-class Classification	Proposed method	**92.96**	**93.88**	**93.97**	**94.57**	**94.77**
	PGLCM-IBL	83.43	89.49	85.52	85.85	88.54
	Difference	9.53	4.39	8.45	8.72	6.23
Binary Classification	Proposed method	**97.16**	**97.68**	**97.98**	**97.88**	**97.79**
	PGLCM-IBL	86.73	91.45	90.17	90.90	90.73
	Difference	10.43	6.23	7.81	6.98	7.06

The bold values indicate the highest accuracy under the same conditions.

**Figure 8:**
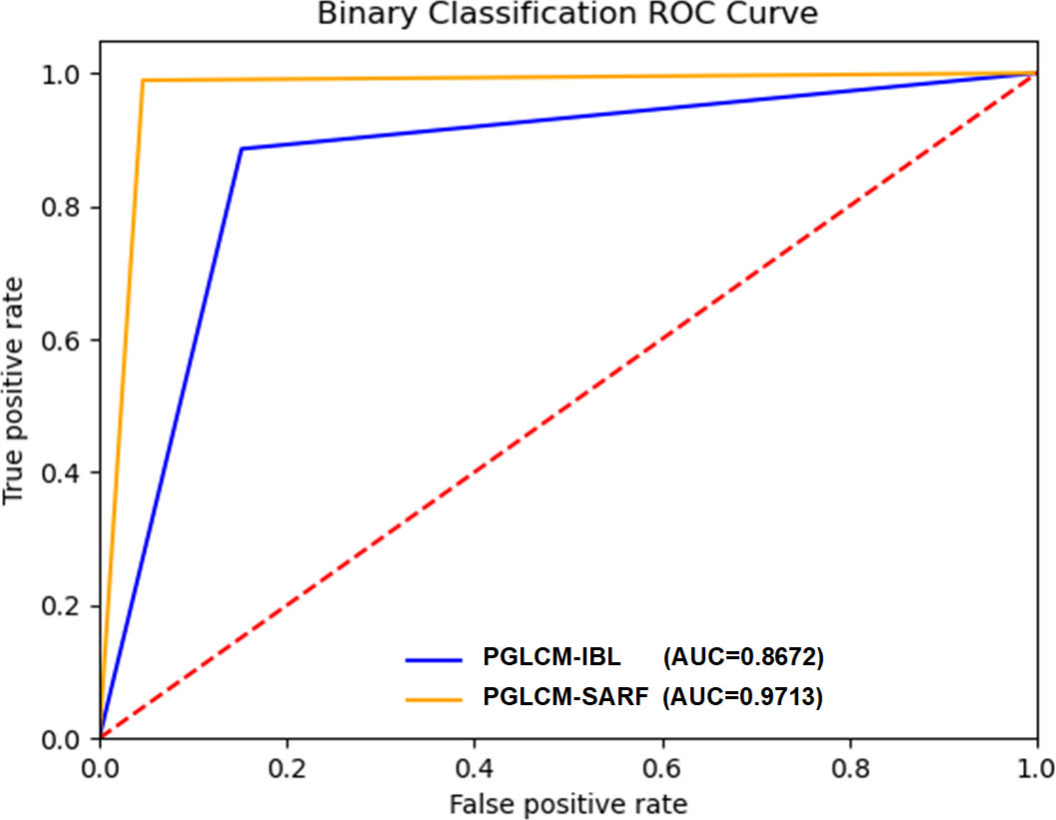
ROC curves of PGLCM-SARF and PGLCM-IBL for binary classification results.

**Figure 9:**
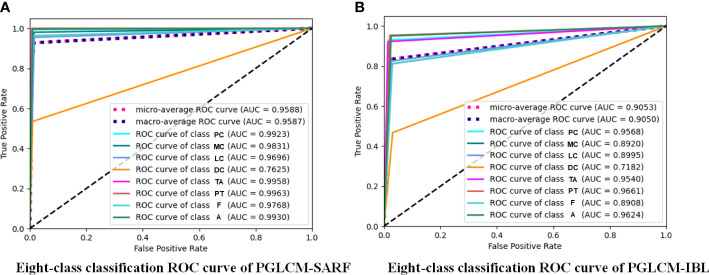
ROC curves of PGLCM-SARF and PGLCM-IBL for eight-class classification results. **(A)** Eight-class classification ROC curves of PGLCM-SARF; **(B)** Eight-class classification ROC curves of PGLCM-IBL.

The ROC curves and AUC values of PGLCM-SARF and PGLCM-IBL classification results are given in **Figures 8** and **9**. As shown in **Figure 8**, the AUC value of PGLCM-SARF for binary classification of breast cancer histopathological images is 0.9713, while that of PGLCM-IBL is only 0.8672, indicating that the proposed model has better binary classification performance. The micro mean AUC value of PGLCM-SARF for eight-class classification of breast cancer histopathological images is 0.9588 as shown in **Figure 9A**, and the micro mean AUC value of PGLCM-IBL is 0.9053 as shown in **Figure 9B**, indicating that the proposed model has higher eight-class classification performance compared to PGLCM-IBL. We found that in **Figure 9A**, our method has the largest AUC value for PT type identification (0.9963) and the smallest AUC value for DC type identification (0.7625), indicating that DC tumor have a certain complexity that makes them difficult to identify.

The comparison results of different models with existing state-of-the-art algorithms under specific magnification conditions are shown in **Table 10**. Among the eight-class classification experiments, literature 286 (23) compared the classificationaccuracy of GoogLeNet, ResNet50 and the Inception-ResNet-V2 proposed in literature (23); literature (28) used CNN for classification of breast cancer histopathological images; literature (29) compared the classification accuracy of PFTAS + QDA, PFTAS + SVM, the PFTAS + RF and Single-Task CNN models. From the experimental results, it can be seen that the proposed PGLCM-SARF achieves 92.96%-94.57% accuracy, which is the highest accuracy compared to all the above models at all 4 magnifications. In the binary classification experiments, literature (30) used a CNN model for classification of breast cancer histopathological images; literature (31) extracted DeCAF features of breast cancer histopathological images for classification; and literature (5) proposed a Single Task CNN model based on magnification independence. From the experimental results, it can be seen that our method achieves an accuracy of 97.16%-97.98%, which is the highest accuracy compared to all the above methods at all 4 magnifications. It is not difficult to find that magnification has a significant impact on the experimental results, due to the fact that the higher the magnification of the medical image, the larger the cellular tissue and the more difficult it is to distinguish the internal environment, leading to poorer results.

**Table 10:** Comparison of classification accuracy (%) of different classification algorithms for BreaKHis dataset.

Category	Model	Magnification			
		40X	100X	200X	400X
Eight-class Classification	GoogLeNet (23)	68.7	65.9	69.1	62.8
	ResNet50 (23)	82.5	78.8	84.3	81
	Inception-ResNet-V2 (23)	86.7	80.3	83.5	68.5
	CNN (28)	88.2	84.6	83.3	84
	CNN (28)	82.70	82.15	83.37	82.40
	PFTAS + SVM (29)	81.65	79.70	85.30	82.30
	PFTAS + RF (29)	81.70	82.60	84.40	81.20
	Single-Task CNN (29)	83.08	84.15	85.67	83.10
	Proposed method	**93.88**	**93.97**	**94.57**	**94.77**
Binary Classification	CNN (30)	89.6	85	84	80.8
	DeCAF features using CNN (31)	84.6	84.8	84.2	81.6
	Single Task CNN (5)	83	83.1	84.6	82.1
	Proposed method	**97.68**	**97.98**	**97.88**	**97.79**

The bold values indicate the highest accuracy under the same conditions.

## 5 Discussion

The experimental results demonstrate that the addition of SAN to RF is advantageous to the adaptive enhancement and refinement of features, which is reflected in the continued improvement of classification accuracy; concurrently, the addition of the GridSearchCV technique to PGLCM-SARF not only avoids the restriction of artificially selected parameters but also contributes to the enhancement of the classification effect. Additionally, from the verification results on BreaKHis dataset demonstrate that our proposed method has superior classification accuracy in comparison to other state-of-the-art algorithms. Despite the clear benefits of the suggested approach, there are still some issues, such as the hardware restriction that the search range of the GridSearchCV only be restricted to the depth of decision trees is 10 to 30 and the number of decision trees is 190 to 230, and the effect of other decision tree parameters on the classification accuracy has not been further studied.

For the sake of verify the universality of the proposed model, we also conduct experiments on MIAS dataset. **Table 11** displays the results of three-class classification on MIAS dataset, where both malignant and benign tumors are accurately identified. Normal pictures are properly recognized in 461 cases, misclassified as malignant in 9 cases, and misclassified as benign in 8 cases. The experimental results demonstrate that our method has more substantial benefits in distinguishing mammographic images from breast cancer histopathological images and has similarly universally applicable on MIAS dataset. To further demonstrate the superiority of the proposed model, comparison results with existing algorithms on MIAS dataset are given in **Table 12**. The literature (32) compared the classification results achieved by using different classifiers and preprocessing method of dataset splitting; the literature (33) proposed a fuzzy multilayer classifier (FMSVM) model; the literature (34) proposed a CNN-based computer detection system, which contains 8 convolutional layers, 4 maximum pooling layers and 2 fully connected layers; literature (35) built a hybrid model based on pulse-coupled neural network (PCNN) and CNN; literature (36) proposed a RANSAC model based on image processing for pectoral muscle detection method and used U-Net architecture to train the model; literature (37) proposed Morph-SPCNN model to solve the limitations of over-segmentation of mammographic images by employing SVM incorporating Gaussian, linear and polynomial kernels as classifiers; literature (38) proposed a texture based associative classifier (TBAC) for automatic breast cancer classification system; literature (39) used halarick’s texture feature extraction algorithm to obtain GLCM from mammographic images, and proposed a three-class classification of mammographic images based on the watershed algorithm combined with the K-NN classifier. By observing the experimental results in **Table 12**, it is easy to find that the recognition results of the proposed algorithm (98.79%) is better than the existing algorithms mentioned above.

**Table 11:** Confusion matrix of three-class classification results and values of evaluation metrics. Sen, sensitivity; Spe, specificity; PPR, positive prediction rate; NPR, negative prediction rate.

Ground Truth	Classification Result			Sen (%)	Spe (%)	PPR (%)	NPR (%)
	Malignant	Benign	Normal				
Malignant	490	0	0	100.00	99.02	98.20	100.00
Benign	0	444	0	100.00	99.17	98.23	100.00
Normal	9	8	461	96.44	100.00	100.00	98.21

((d, n) = (20, 204)).

**Table 12:** Comparison of the classification accuracy of different classification algorithms for MIAS dataset.

Model	Accuracy(%)
VGG19+SVM(27)	95.92
ResNet50+SVM (27)	96.87
Inception-V2&ResNet+SVM (27)	94.76
Inception V3+SVM (27)	98.45
FMSVM (14)	98.50
CNN (12)	92.54
PCNN-CNN (1)	98.77
RANSAC (17)	92.20
Morph-SPCNN (26)	87.80
TBAC (8)	94.66
Watershed+K-NN (22)	85.00
Proposed method	**98.79**

The bold values indicate the highest accuracy.

**Figure 10:**
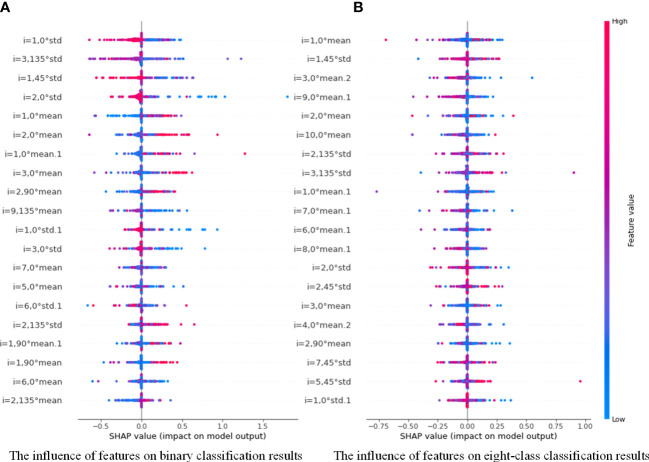
Distribution of the influence of different features on PGLCM-SARF model.0 **(A)** The influence of features on binary classification results; **(B)** The influence of features on eight-class classification results.

Compared with the poor interpretability of deep learning, the proposed model in this paper has significant advantages. For example, we can draw a scatter plot of our feature density with the help of the model interpreter SHAP, as shown in **Figure 10**. SHAP is a game theory-based approach to interpreting machine learning models, as proposed by lundberg and lee (40). The core idea of SHAP is to calculate the marginal contribution of features to the model output, and then interpret the “black box model” at both global and local levels. During model training or testing, a corresponding prediction value is generated for each sample, and the SHAP value is the corresponding value attributed to each feature in the sample (41). In the figure, the horizontal axis represent the SHAP values (the distribution of feature effects on the model output) and the vertical axis represent the feature ranking corresponding to the sum of the 400 sample SHAP values. Each point represents a sample, with thesample size stacked vertically, and the colors denoting the feature values (red corresponding to high values and blue corresponding to low values). For instance, the first row of **Figure 10A** shows that high “i=1, 0°std” (meaning the standard deviation values of the contrast, correlation, energy and homogeneity features at a distance of 1 and in the 0° direction) (red) has a negative effect on the prediction, while low “i=1, 0°std” (blue) has a positive effect on the prediction. Therefore, through the information in the figure, we can analyze the influence of different types of features on the proposed model, and provide theoretical basis for further optimization of the model in the future.

## 6 Conclusion

In this paper, a breast cancer image recognition method based on PGLCM-SARF was proposed and its performance was evaluated on two datasets (BreaKHis and MIAS). Our overall framework was composed of feature extraction and classification. In the feature extraction stage, the multi-scale fusion features of images were extracted using PGLCM, and feature representation capability by introducing Gaussian pyramid technique. In the classification stage, the proposed SARF model achieved adaptive refinement and enhancement of features to achieve higher classification accuracy by introducing SAN. In the meantime, the GridSearchCV technique was used to optimize the classification ability of SARF, which effectively avoided the limitation of artificial parameter selection. Experiments showed that, compared to other state-of-the-art algorithms, the performance of our method had been significantly improved on histopathological image dataset (BreakHis). In addition, our method was also applicable to mammographic image dataset (MIAS), which reflected the universality of the model. Furthermore, using the model interpretable SHAP, we analyzed the degree of influence of each scale feature in the sample on the prediction results of SARF, which played an active role in the subsequent model optimization.

## Data availability statement

The original contributions presented in the study are included in the article/supplementary material. Further inquiries can be directed to the corresponding author.

## Author contributions

Conceptualization and Formal analysis—JL, JS, Data curation—JS, JC. Writing-Original Draft-JC, JS, LH, Writing—Edit and Review-JL, ZD. All authors contributed to the article and approved the submitted version.
